# Causal effect of physical activity and sedentary behaviors on the risk of osteoarthritis: a univariate and multivariate Mendelian randomization study

**DOI:** 10.1038/s41598-023-46984-2

**Published:** 2023-11-08

**Authors:** Xingzhao Li, Sibo Wang, Wanguo Liu, Han Wu, Yuhang Zhu

**Affiliations:** 1 Department of Ultrasound, China-Japan Union Hospital of Jilin University, Changchun, China; 2 Department of Neurology, Center for Neuroscience, The First Hospital of Jilin University, Changchun, China; 3 Department of Orthopedics, China-Japan Union Hospital of Jilin University, Changchun, 130033 China

**Keywords:** Osteoarthritis, Epidemiology, Lifestyle modification, Genome-wide association studies, Genetic association study, Risk factors

## Abstract

There is still a lot of ambiguity about the link between physical activity (PA), sedentary behaviors (SBs) and osteoarthritis (OA). This study aimed to investigate the causal relationship of PA/SBs on the risk of OA. A univariate and multivariate Mendelian randomization (MR) analysis was conducted to investigate the causal effect of five PA phenotypes and three SB phenotypes on overall OA, knee OA, hip OA, total hip arthroplasty, and total knee arthroplasty (TKA). MR methods used were inverse-variance weighting, MR-Egger regressions, and weighted median. Sensitivity analysis examined horizontal pleiotropy and heterogeneity, and confirmed the reliability of the results. After false discovery rate, light do-it-yourself (DIY) activities decreased the risk for overall OA (OR: 0.32, 95% CI 0.16–0.65), and knee OA (OR: 0.26, 95% CI 0.12–0.51). Resulting in a decreased risk of walking for pleasure on overall OA (OR: 0.87, 95% CI 0.70–1.04) and knee OA (OR: 0.14, 95% CI 0.06–0.32) was also observed. Television viewing, however, significantly increased the risk of OA, knee OA, hip OA, and TKA. MVMR findings revealed independent causal impacts of walking for pleasure and watching television on overall and knee OA, taking into account BMI, smoking, and education. This study suggested that light DIY and walking for pleasure were beneficial for preventing OA, and the risk of OA and TKA increased with prolonged television watching.

## Introduction

The most common degenerative joint disease for the aging population, osteoarthritis (OA), ranks fifth in global disability causes^1^. The number of people suffering from OA worldwide exceeds 300 million, especially knee and hip OA^2^. In spite of the fact that OA risk factors, including female sex, obesity, joint injuries, and high bone mineral density, it remains a priority to study modifiable variables that can prevent OA's pain and impairment^3^.

As a public health intervention, many major diseases can be treated through physical activity (PA), including cardiovascular disease, diabetes, and obesity^4,5^. However, there is still a lot of ambiguity about the link between PA and OA. Although evidence suggests that PA such as strengthening exercises and aerobic walking, is beneficial for reducing pain and improving the physical function of OA patients^6,7^, studies have shown that high levels of PA can lead to knee structural damage and increase the risk for knee arthroplasty^8,9^. Moreover, a Danish trial revealed that the improvement of exercise after 9 weeks was also similar, with no essential difference in comparison with the placebo^10^. The contextual effects seem to explain essentially all the improvement after an exercise intervention^11^. On the other hand, sedentary behaviors (SBs), such as sitting for prolonged periods, could increase the risk of OA^12^. Nevertheless, it is likely that observational studies were confounded by inverse causality or confounding variables, which potentially results in biased associations and conclusions. Therefore, it is unclear how PA and SBs link to OA and whether or not there are separate causal associations.

In order to discover single nucleotide polymorphisms (SNPs) associated with common complex diseases, genome-wide association studies (GWAS) have been greatly helpful^13^. It can comprehend the genetic underpinnings of several complicated features in widespread human illnesses. Genetic variants are used as instrumental variables (IV) in Mendelian randomization (MR) to examine risk variables and disease outcomes^14^. It is possible to exclude confounding factors and identify causal factors of a specific outcome by MR analysis since genetic variants are assigned randomly before disease onset^15^.

In this study, a particular focus was placed on exploring the potential causal effects of five phenotypes of PA and three phenotypes of SBs on different OA subtypes and relevant arthroplasty using large-scale GWAS data. As a result, this study assessed the impact of physical activity on OA, identified a potentially susceptible sedentary population, and provided recommendations for OA prevention.

## Methods

### Study design overview

**Figure 1:**
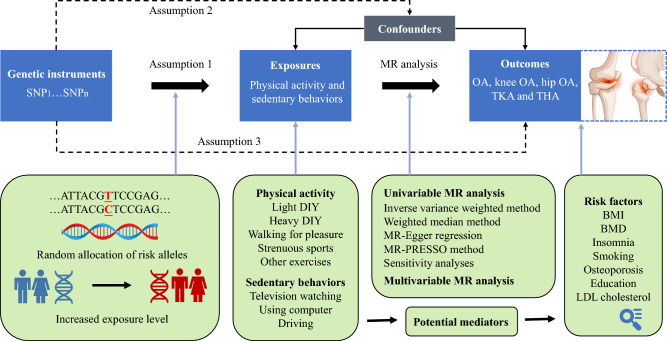
Study design overview. OA, osteoarthritis; TKA, total knee arthroplasty; THA, total hip arthroplasty; SNP, single nucleotide polymorphism; MR, Mendelian randomization; MR-PRESSO, Mendelian Randomization Pleiotropy RESidual Sum and Outlier.

This MR study was designed as shown in Fig. 1. To examine the causal effects, a univariable MR analysis (UVMR) was performed between PA/SBs and OA/TJA using GWAS summary statistics first, then potential mediators correlated with PA/SBs were evaluated in the risk factors analysis, and validated by multivariable MR analysis (MVMR). The random distribution of SNPs in offspring is mimicked by this instrumental-variable analysis similar to RCT. Only genetic variants that meet the following three requirements can be considered IVs^16^. As noted above, genetic variants have a strong correlation with exposure. As well as this, genetic variants don't correlate with confounders. Lastly, genetic variants cannot directly influence outcomes. STROBE-MR guidelines were followed for the analysis^17^.

### Data sources and instrumental variable selection

The UK Biobank is a large prospective cohort study including half a million volunteer participants in the United Kingdom aged between 40 and 69 years. The UK Biobank has genotyped the DNA from participants' blood samples. This genetic data provides information about participants' genetic variations, which can be linked to various health outcomes. Summary-level GWAS from the UK Biobank of 460,376 participants was used to construct instrumental variables for PA^18,19^, which included five phenotypes: light do-it-yourself (DIY) (eg., pruning, watering the lawn), heavy DIY (eg., weeding, lawn mowing, carpentry, digging), walking for pleasure, strenuous sports, and other exercises. At the time of enrollment, data on PA were gathered using items modified from the well-validated International Physical Activity Questionnaire (IPAQ). Participants, where appropriate, indicated their participation (yes or no), frequency (days/week), and/or length (minutes/day) for each specified physical activity. Full details of instrumental variable selection are given in Appendix 1. Finally, 12, 18, 20, 6, and 15 SNPs were used as IVs for light DIY, heavy DIY, walking for pleasure, strenuous sports, and other exercises in our study, respectively.

From the UK Biobank's latest summary-level GWAS of 422,218 participants, we identified candidate genetic instruments associated with SBs^20^. Answers to the questions “How many hours do you spend watching TV/driving/using the computer (Do not include using a computer at work) in a typical day?” were used to calculate the amount of time participants spent on each behavior. GWAS meta-analyses included three categories of IVs for SBs: television watching, computer use, and driving. After adjusting covariates, 151, 47, and 5 SNPs were used as IVs for television watching, computer use, and driving.

From the latest publicly available GWAS with up to 826,690 participants from the Genetics of Osteoarthritis (GO) Consortium, we compiled summary statistics for overall OA, knee OA, hip OA, total knee arthroplasty (TKA), and total hip arthroplasty (THA)^21^. Based on the TREAT-OA consortium's definition, OA cases were defined based on self-reported, hospital diagnoses, ICD10 codes, or radiographic findings. Variants were grouped according to which OA site they were associated with. A detailed list of SNPs for PA/SBs on OA and TJA is presented in Supplementary file 1, Tables S1–S5.

### Univariable MR analysis

In this study, Two-sample MR (version 0.5.6) and MRPRESSO (version 1.0) packages with R software (version 4.2.2) were used for UVMR. We estimated the causal effects of genetically predicted exposure on the outcome using the inverse-variance-weighted (IVW) method as our primary MR analysis method^22^. Additionally, MR-Egger regression and weighted median method were implemented in addition to the IVW, since these methods can provide more comprehensive estimates^23,24^. IVW analyses were conducted using a false discovery rate (FDR) adjusted *P*-value (also known as q-value) for testing multiple hypotheses. A significance level of less than 0.05 was set for causal inference. Additionally, binary outcomes were used to calculate the post-hoc power using an online power calculation (https://shiny.cnsgenomics.com/mRnd/).

### Sensitivity analysis

When genetic variations linked to exposure of interest have a direct impact on the result via multiple pathways other than the expected exposure, this is known as horizontal pleiotropy^25^. To assess whether the results were robust and pleiotropic, we performed Cochran's Q statistics, MR-Egger intercept tests, funnel pot analyses, and leave-one-out analyses^25^. In particular, Cochran Q tests were used to detect heterogeneity if the *P* value is less than 0.05. Additionally, we evaluated horizontal pleiotropy using the intercept term derived from MR-PRESSO^26^. Moreover, the Steiger test was utilized to validate whether the observed causalities were biased owing to reversed causation.

### Multivariable MR analysis

MVMR extends UVMR by including all factors in the same model and estimating their causal effects jointly on OA risk^27^. Three potential mediators (BMI^28^, smoking^29^, and education^30^) that had strong genetic correlations with PA/SBs were chosen. In the MVMR analysis, the mediation effect of these exposures was validated simultaneously, and the mediation effect of single exposures was estimated separately. To infer causal effects in MVMR, weighted linear regression-based IVW and MR-Egger approaches were used. MVMR was carried out by Mendelian Randomization (version 0.7.0) and MVMR (version 0.3.0) packages.

### Ethics approval and consent to participate

This manuscript does not include clinical studies or patient data. All data is exchanged from public open databases thus avoiding ethical disputes.

## Results

### Univariable MR analysis

**Figure. 2:**
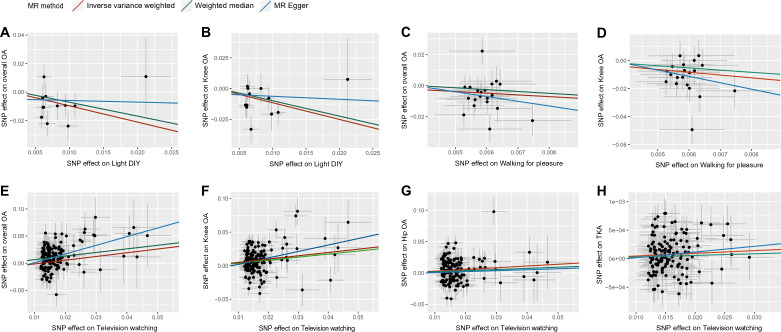
Scatterplots showing the effect of SNPs on the outcome (y-axis) and exposure (x-axis) with significant effect sizes with 95% confidence intervals. Slopes represent estimates for three different MR modes. Radiation SNP effects are plotted as standard deviation (SD) per unit and outcomes (total OA, knee OA, hip OA, and TKA) are plotted as log probability per unit of expression. (**A**, **B**) the association between light DIY and the risk of overall OA and knee OA; (**C**, **D**) the association between walking for pleasure and the risk of overall OA and knee OA; (**E**, **H**) the association between television watching and risk of overall OA, knee OA, hip OA, and TKA. SNPs, single nucleotide polymorphisms; MR, Mendelian randomization; OA, osteoarthritis; TKA, total knee arthroplasty.

As shown in Table 1, eight out of forty exposure-outcome associations retained significant and determined to be causal effects connected with overall OA, knee OA, hip OA, and TKA, after FDR adjustment. A causal association was found between light DIY and overall OA (OR: 0.319, 95% CI 0.158–0.646, q = 0.011), and knee OA (OR: 0.258, 95% CI 0.116–0.570, q = 0.008). In addition, walking for pleasure was associated with a decreasing risk of overall OA (OR: 0.869, 95% CI 0.702–1.042, q = 0.016), and knee OA (OR: 0.136, 95% CI 0.059–0.318, q = 5.65 × 10^–5^). In contrast, a significant association was found between genetically proxied television watching and overall OA (OR: 1.988, 95% CI 1.637–2.413, q = 1.44 × 10^–10^), knee OA (OR: 1.637, 95% CI 1.396–1.920, q = 2.66 × 10^–8^), hip OA (OR: 1.318, 95% CI 1.110–1.565, q = 0.011), and TKA (OR: 1.104, 95% CI 1.001–1.207, q = 0.021). Additionally, genetic liability was not associated with heavy DIY, strenuous sports, other exercises, computer use, or driving on the risk of OA and TJA. The scatter plots (Fig. 2A-H) showed a significant causal relationship between PA/SBs and OA/TJA risk.

**Table 1:** Significant MR estimates of the causal association between PA/SBs on OA and total joint arthroplasty.

Exposure	Outcome	Nsnp	IVW			Weighted median		MR Egger	
			OR (95CI)	*P* value	q value	OR (95CI)	*P* value	OR (95CI)	*P* value
Light DIY	Overall OA	12	0.319 (0.158–0.646)	0.001	0.011	0.377 (0.156–0.906)	0.029	0.237 (0.021–2.654)	0.261
Light DIY	Knee OA	12	0.258 (0.116–0.570)	8.18 × 10^–4^	0.008	0.288 (0.096–0.861)	0.025	0.785 (0.042–14.528)	0.874
Walking for pleasure	Overall OA	20	0.869 (0.702–1.042)	0.003	0.016	0.872 (0.730–1.049)	0.469	1.061 (0.761–1.480)	0.736
Television watching	Overall OA	151	1.988 (1.637–2.413)	3.60 × 10^–12^	1.44 × 10^–10^	1.937 (1.476–2.543)	1.90 × 10^–6^	4.924 (2.041–11.879)	5.19 × 10^–4^
Television watching	Knee OA	151	1.637 (1.396–1.920)	1.33 × 10^–9^	2.66 × 10^–8^	1.580 (1.334–1.872)	1.18 × 10^–6^	2.616 (1.255–5.450)	0.012
Television watching	Hip OA	151	1.318 (1.110–1.565)	0.001	0.011	1.199 (0.973–1.478)	0.036	1.167 (0.527–2.584)	0.703
Television watching	TKA	147	1.104 (1.001–1.207)	0.004	0.021	1.102 (0.998–1.207)	0.179	1.012 (0.991–1.028)	0.279

*MR* Mendelian randomization, *OA* osteoarthritis, *TKA* total knee arthroplasty, *IVW* inverse variance weighted, *OR* odds ratio, *CI* confidence interval; q value, FDR-adjusted *P* value.

### Sensitivity analysis

As shown in Table 2, according to the MR-Egger intercept test, all *P* values were greater than 0.05, thus indicating no horizontal pleiotropy. Analyzing the Q test, heterogeneity was observed between walking for pleasure and overall OA, and television watching and overall OA, Hip OA, and TKA (*P* < 0.05). The global test of MR-PRESSO eliminated horizontal pleiotropy, indicating that MR estimates were not biased in the context of heterogeneity. All the Steiger *P* values were less than 0.05, suggesting that no reverse causality bias was found in the identified causalities. Moreover, the estimation was unaffected by any SNP with a substantial impact size, according to the leave-one-out test (Supplemental Figs. 1–2). Forest plots (Supplementary Figs. 3–4) were observed in the MR-SingleSNP test for causal effects of causal PA/SBs associated with single SNPs. Funnel plots were symmetrical, indicating that the estimates were not violated (Supplemental Fig. 5).

**Table 2:** Sensitivity analysis of the causal associations between PA/SBs on OA and total joint arthroplasty.

Exposure	Outcome	Nsnp	Cochran's Q test		MR-Egger regression		MR PRESSO		Steiger test	
			Q	*P* value	Egger intercept	*P* value	Global.Test	*P* value	Direction	*P* value
Light DIY	Overall OA	12	22.964	0.191	0.008	0.148	25.886	0.341	True	7.23 × 10^–7^
Light DIY	Knee OA	12	22.102	0.485	0.002	0.451	24.778	0.597	True	6.25 × 10^–3^
Walking for pleasure	Overall OA	20	50.437	1.19 × 10^–8^	− 0.001	0.343	71.158	0.124	True	1.72 × 10^–4^
Walking for pleasure	Knee OA	20	13.583	0.226	0.004	0.602	18.366	0.316	True	5.02 × 10^–3^
Television watching	Overall OA	151	248.971	6.86 × 10^–7^	0.002	0.758	26.434	0.112	True	5.77 × 10^–4^
Television watching	Knee OA	151	174.243	0.085	− 0.012	0.146	19.671	0.149	True	1.48 × 10^–4^
Television watching	Hip OA	151	339.631	9.86 × 10^–17^	− 0.007	0.201	35.474	0.071	True	1.17 × 10^–4^
Television watching	TKA	147	209.871	6.18 × 10^–5^	− 8.46 × 10^–5^	0.558	22.991	0.121	True	7.32 × 10^–3^

*MR* Mendelian randomization, *OA* osteoarthritis, *TKA* total knee arthroplasty, *IVW* inverse-variance weighted, *MR PRESSO* MR Pleiotropy RESidual Sum and Outlier, *OR* odds ratio, *CI* confidence interval.

**Figure. 3:**
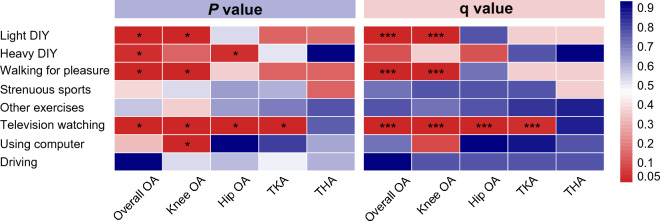
A panorama of all PA/SB causality estimates for osteoarthritis and total joint arthroplasty, determined according to the nominal significance of *P* values and q-values (FDR-adjusted). PA, physical activity; SBs, sedentary behaviors; OA, osteoarthritis; TKA, total knee arthroplasty; THA, total hip arthroplasty; FDR, false discovery rate. * and *** represent the significance of the *P* value and q value, respectively.

A summary of all the causal effects can be seen in Fig. 3. and Supplementary file 2, Table S6. Summary data of sensitivity analysis of all the MR assumptions were indicated in Supplementary file 2, Table S7. Except for television watching to TKA, all statistical powers of significance were greater than 60% (Supplementary file 2, Table S8), and the *F* statistic for each exposure exceeded the empirical threshold of 10 (Supplementary file 1, Tables S1–S5). Additionally, each SNP was checked in Phenoscanner's genetic instrument to assess whether prospective risk factors violated it (Supplementary file 2, Table S9). We did not detect any potential risk factors that compromised the robustness of our causality estimations.

### Multivariable MR analysis

**Figure. 4:**
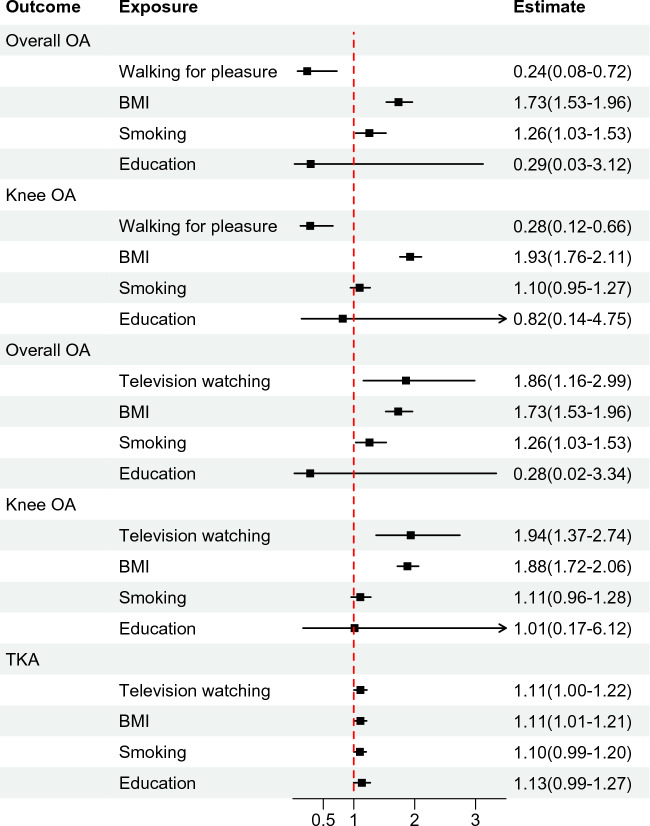
Forest plots of multivariate Mendelian randomization analyses examined causal associations between PA/SB and OA and arthroplasty, adjusted for BMI, smoking status, and education. Estimates of causality were expressed as odds ratios (ORs) and 95% confidence intervals (CIs). PA, physical activity; SBs, sedentary behaviors; OA, osteoarthritis; TKA, total knee arthroplasty.

In MVMR analysis, BMI, smoking, and education were taken into consideration to determine whether PA/SBs were associated with OA in an independent causal manner. Walking for pleasure had an independent causal effect on overall OA (OR: 0.235, 95% CI 0.076–0.723), and knee OA (OR: 0.284, 95% CI 0.123–0.657). Moreover, television watching was strongly associated with a directed causal effect on OA risk (OR: 1.858, 95% CI 1.157–2.986), knee OA (OR: 1.936, 95% CI 1.367–2.742), and TKA (OR: 1.109, 95% CI 1.002–1.216) (Fig. 4). No causal association was found between light DIY and OA, and watching TV and hip OA. The estimated effects between IVW and Egger analysis were similar, and MVMR model estimates were not distorted by directional pleiotropy since all *P* values for MVMR-Egger intercept tests exceeded thresholds of 0.05 (Supplementary file 2, Table S10).

## Discussion

The present study is the first to comprehensively and deeply examine the causal effects of PA/SBs on OA and TJA using GWAS summary-level data. To identify causal effects, multivariate and univariate MR analyses were conducted on 40 potential associations and found that light DIY, walking for pleasure, and television watching were associated with the risk of OA and TJA.

Previous studies indicated that controversy still existed on the relationship between PA and OA^7–9,31,32^. According to the current study, increased levels of light DIY and walking for pleasure were associated with an increased risk of OA in both the knee and overall. Although the results from other patterns like heavy DIY, strenuous sports, and other exercises showed a consistent trend, nonsignificant causal associations were detected. Several studies have linked PA with OA, consistent with our findings. Chang et al.^31^ followed 1194 participants for over 10 years in a cohort study and reported no association between long-term strenuous PA and knee OA. The study also indicated that a low-to-moderate level of PA displayed a protective effect on knee OA. Bell et al.^33^ conducted a systematic review of 28 RCTs with 2,789 participants and indicated that people with knee OA may benefit from walking and mixed exercise, but not from resistance training. A systematic review and population modeling study found that even relatively modest intensity bouts of PA could help maintain or improve the life quality of OA patients compared with typical levels of sedentariness^34,35^. In cohort studies of healthy adults, PA at low or moderate intensities improved cartilage, thereby possibly preventing OA degeneration^36,37^. Although it has been suggested that high levels of PA in patients with OA may damage articular joint cartilage and increase the risk for TJA, it is unclear whether such structural damage actually causes OA^38^. We hypothesize that the potential damage of high levels of PA dampens its beneficial effects on OA, which weakened the causal relationship of heavy DIY and strenuous sports with OA in this literature. The underlying mechanism linking different types of PA to hip/knee OA remains inclusive, which is therefore necessary to further examine.

Television watching was shown to be a risk factor for OA, knee OA, hip OA, and TKA in this study by robust genetic evidence. Notably, we think that OA was induced by the sedentary behavior caused by TV viewing, not the TV watching itself. SBs and OA have been associated in previous observational studies^12,39,40^. These studies specifically demonstrated that sedentary behavior could increase the risk of developing knee and hip OA, and reducing sedentary time helped OA patients feel less pain and exhaustion, which in turn enhanced their quality of life. In comparison to other sedentary behaviors, there is evidence that watching leisure television leads to an increase in food intake and energy intake as well as a decrease in physical activity^41,42^. It is possible that watching television contributes to a surplus of energy by decreasing rest time, lowering energy expenditure, and consuming too much energy^41^. Additionally, watching television is a less cognitive and conversational form of leisure and entertainment than using the computer or driving^42^. Further, television watching specifically impacts the mental and physical health of individuals (such as anxiety and sadness)^43^. Hence, television watching, as a unique sedentary behavior, affects the development of OA in a specific way.

Prior MR analysis indicated that several risk factors might affect these causal inferences. Smoking status plays a significant role in the development of OA, and has been indicated as an independent deleterious causal effect upon OA by MR analysis^44^. Substantial evidence suggested that OA burden and TKA costs were likely to increase significantly with obesity^45^. Higher education was also substantiated as a protective factor for OA^30^. However, these potential mediators need to be further validated in multivariate MR analysis. Taking the effects of these potential mediators into consideration, MVMR results showed that the independent causal effects of walking for pleasure and television watching suggested the direct genetic associations with overall OA and knee OA, which had been overlooked in the previous studies. After adjustment for potential mediators, light DIY displayed no causal association with overall OA and knee OA, which implies that the causality between light DIY and OA may be mediated by smoking and education. Even though MVMR can assess PA/SBs' unconfounded effect on OA risk, the degree to which BMI, smoking, and education affect this effect cannot be determined. Consequently, the precise degree of crucial intermediate elements in PA/SBs for OA and TJA needs to be explored in further studies.

In comparison with observational studies, the primary advantage of this study is the use of an MR design, which is less susceptible to confounding and does not suffer from time-related bias. STROBE-MR guidelines were strictly followed in this MR study. Our study examined genetically predicted exposures to OA with data from the largest summary-level dataset available for this disease. Demographic stratification was eliminated by restricting the included participants to the European population. A variety of methods were also applied in our study, including sensitivity analyses and MVMR analyses, to improve the reliability of causal conclusions.

The MR approach has several limitations. First, age and gender are known to be significant risk factors for OA. It is difficult, however, to establish any nonlinear relationships or stratification effects using summary-level data. Second, the summary GWAS data only included European individuals, making it difficult to explain our findings across the entire population. Third, we were not able to measure PA/SBs by metabolic equivalent (MET) because there were no relevant databases. The causality link between PA/SBs and OA/TJA needs to be further confirmed, and potential mechanisms need to be explored to develop relevant clinical recommendations.

## Conclusion

Overall, the research suggests that light DIY and walking for pleasure are important factors in the prevention of OA, while sedentary behavior caused by television watching may increase the risk of OA and TKA. People who are at risk for osteoarthritis should aim to engage in a regularly low-to-moderate level of PA, and try to reduce television watching time. This research examines the potential causal relationship between PA/SBs and OA/TJA, and may help identify new treatment and rehabilitation strategies to address this condition.

### Supplementary Information

Supplementary Information 1.

Supplementary Information 2.

Supplementary Information 3.

Supplementary Information 4.

Supplementary Information 5.

Supplementary Information 6.

Supplementary Information 7.

Supplementary Information 8.

Supplementary Information 9.

Supplementary Figures.

## Data Availability

The information utilized to produce the study's findings of osteoarthritis was gathered from the publicly available Genetic Consortium genome association from the website https://msk.hugeamp.org/downloads.html. The data on physical activity and sedentary behaviors were downloaded from the UK biobank from the website https://www.ukbiobank.ac.uk/. The data upon which this paper is based can be found in this paper and its online supplement.

## Abbreviations

GWAS: Genome-wide association studies
SNP: Single nucleotide polymorphism
IV: Instrumental variable
MR: Mendelian randomization
IVW: Inverse variance weighted
MR-PRESSO: Mendelian Randomization Pleiotropy RESidual Sum and Outlier
PA: Physical activity
SBs: Sedentary behaviors
OA: Osteoarthritis
DIY: Do-it-yourself
TKA: Total knee arthroplasty
THA: Total hip arthroplasty
TJA: Total joint arthroplasty
FDR: False discovery rate
BMI: Body mass index
BMD: Bone mineral density
