# Supplementary material for: Causal effect of physical activity and sedentary behaviors on the risk of osteoarthritis: a univariate and multivariate Mendelian randomization study
Source: Sci Rep. 2023 Nov 8;13:19410. doi: 10.1038/s41598-023-46984-2 (PMC10632381; doi:10.1038/s41598-023-46984-2)
Supplement: Supplementary file 3 — Supplementary Information 3. [file 41598_2023_46984_MOESM3_ESM.docx]

**Appendix 1**

The genetic instruments were selected using the following criteria: i) GWAS correlation *P* value of 5 × 10^–8^, ii) LD r^2^ threshold of 0.001, and window size of one MB from the IV database. A further assessment of the strength of IVs was conducted by calculating R^2^ and F-statistics using the exposure dataset, number of IVs, and genetic variance. Moreover, Phenoscanner database was used to search all SNPs associated with exposure in order to identify confounding factors. The SNPs were manually removed to avoid the possibility of pleiotropic effects.
