# Supplementary figures and images for: Causal effect of physical activity and sedentary behaviors on the risk of osteoarthritis: a univariate and multivariate Mendelian randomization study
Source: Sci Rep. 2023 Nov 8;13:19410. doi: 10.1038/s41598-023-46984-2 (PMC10632381; doi:10.1038/s41598-023-46984-2)

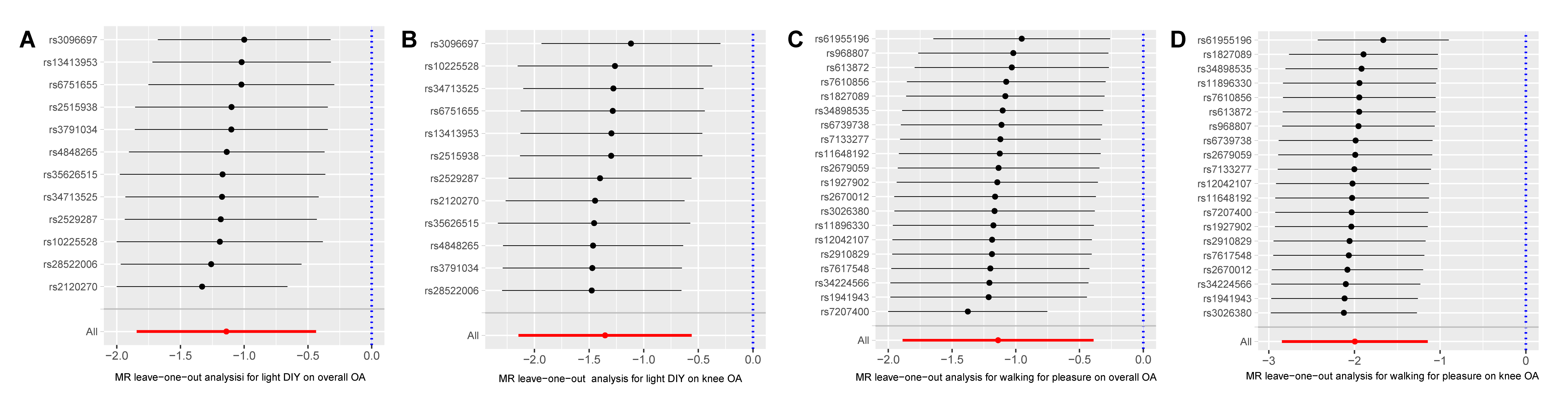

Supplement: Supplementary file 5 — Supplementary Information 5. [file 41598_2023_46984_MOESM5_ESM.tif]

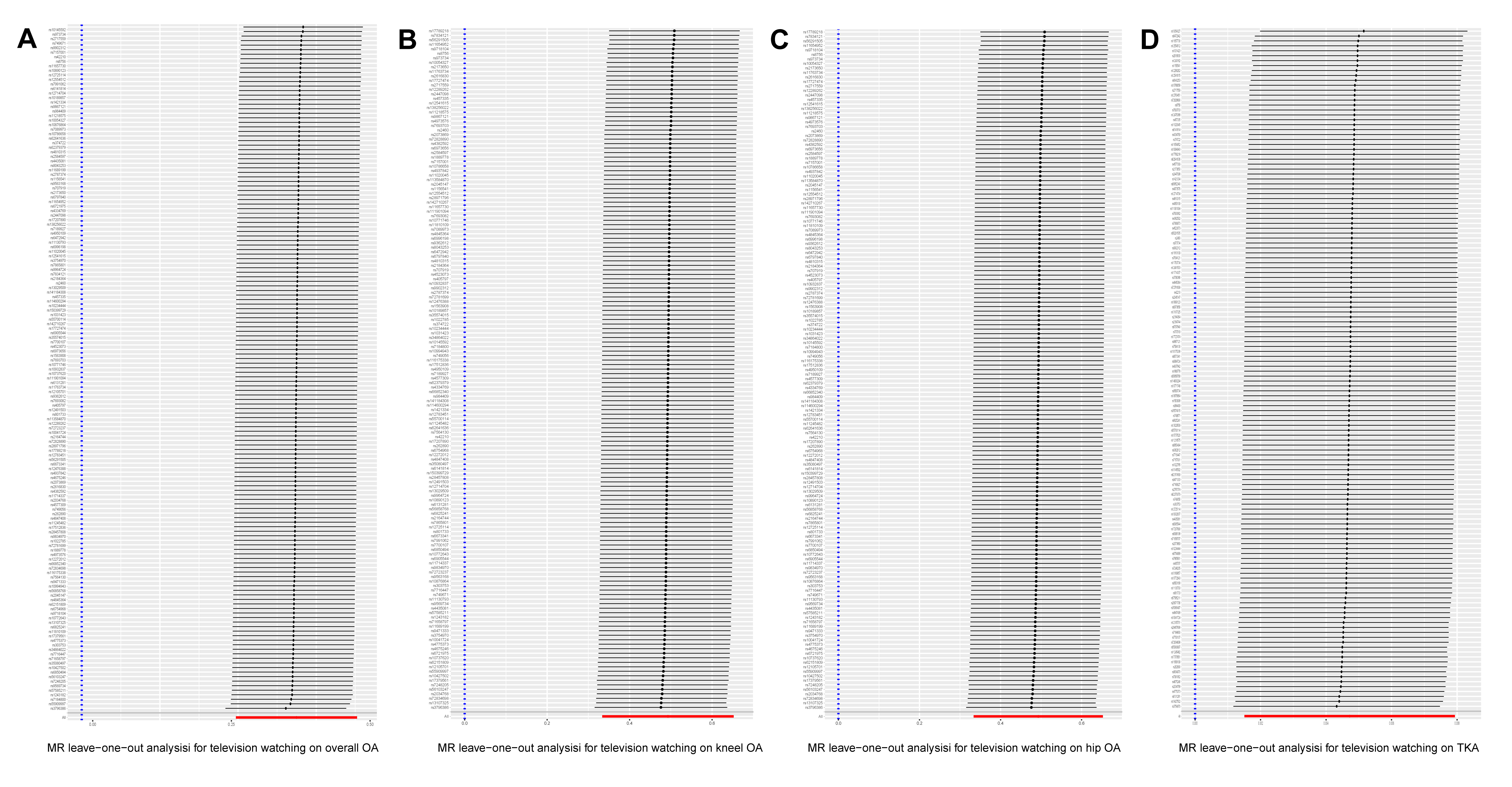

Supplement: Supplementary file 6 — Supplementary Information 6. [file 41598_2023_46984_MOESM6_ESM.tif]

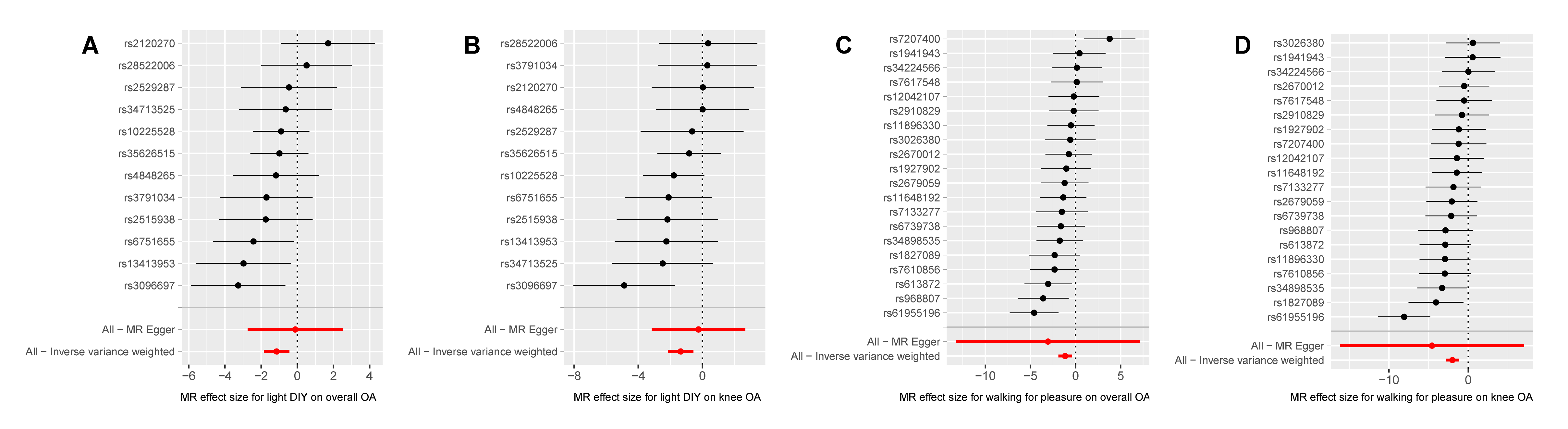

Supplement: Supplementary file 7 — Supplementary Information 7. [file 41598_2023_46984_MOESM7_ESM.tif]

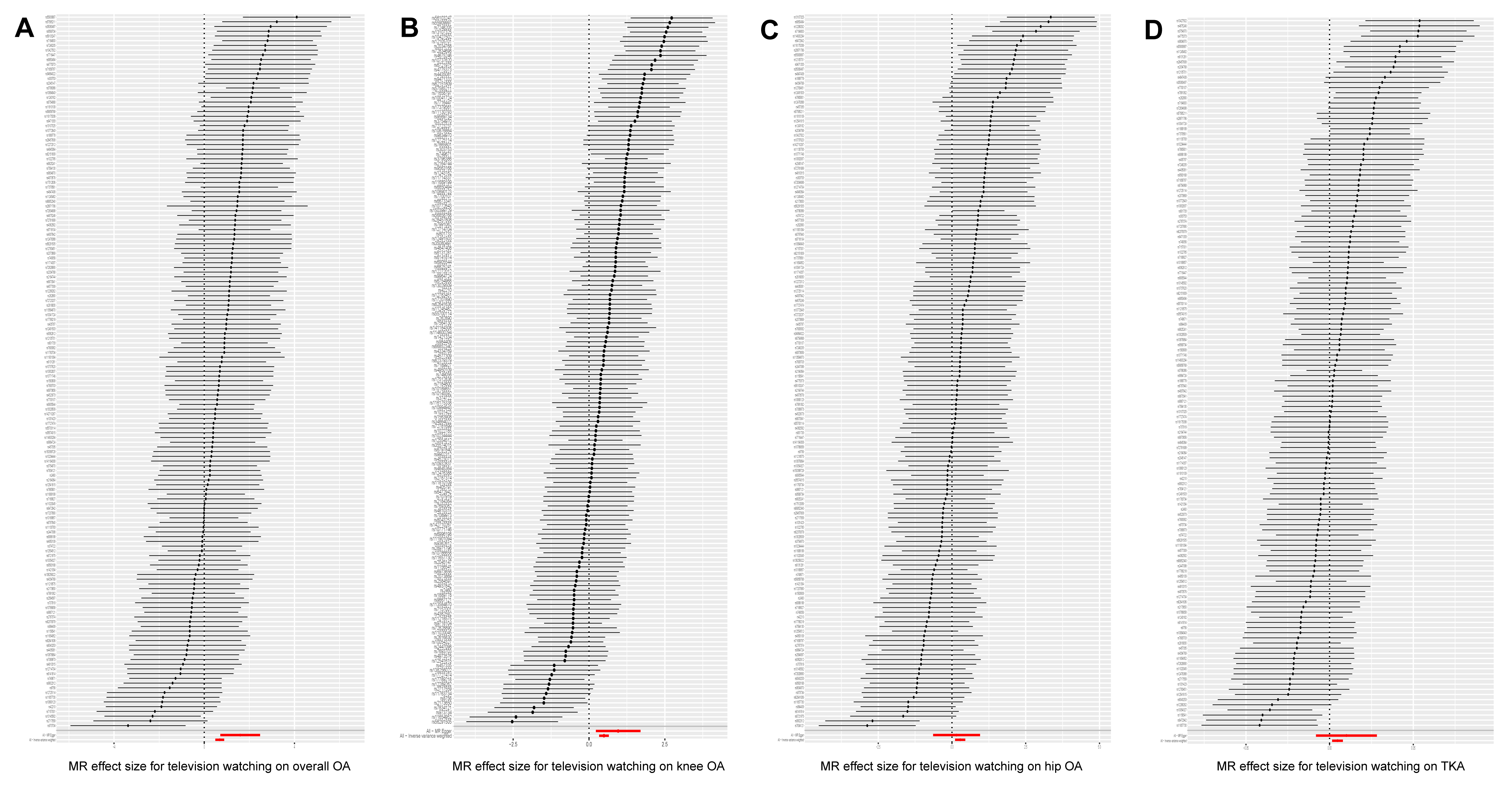

Supplement: Supplementary file 8 — Supplementary Information 8. [file 41598_2023_46984_MOESM8_ESM.tif]

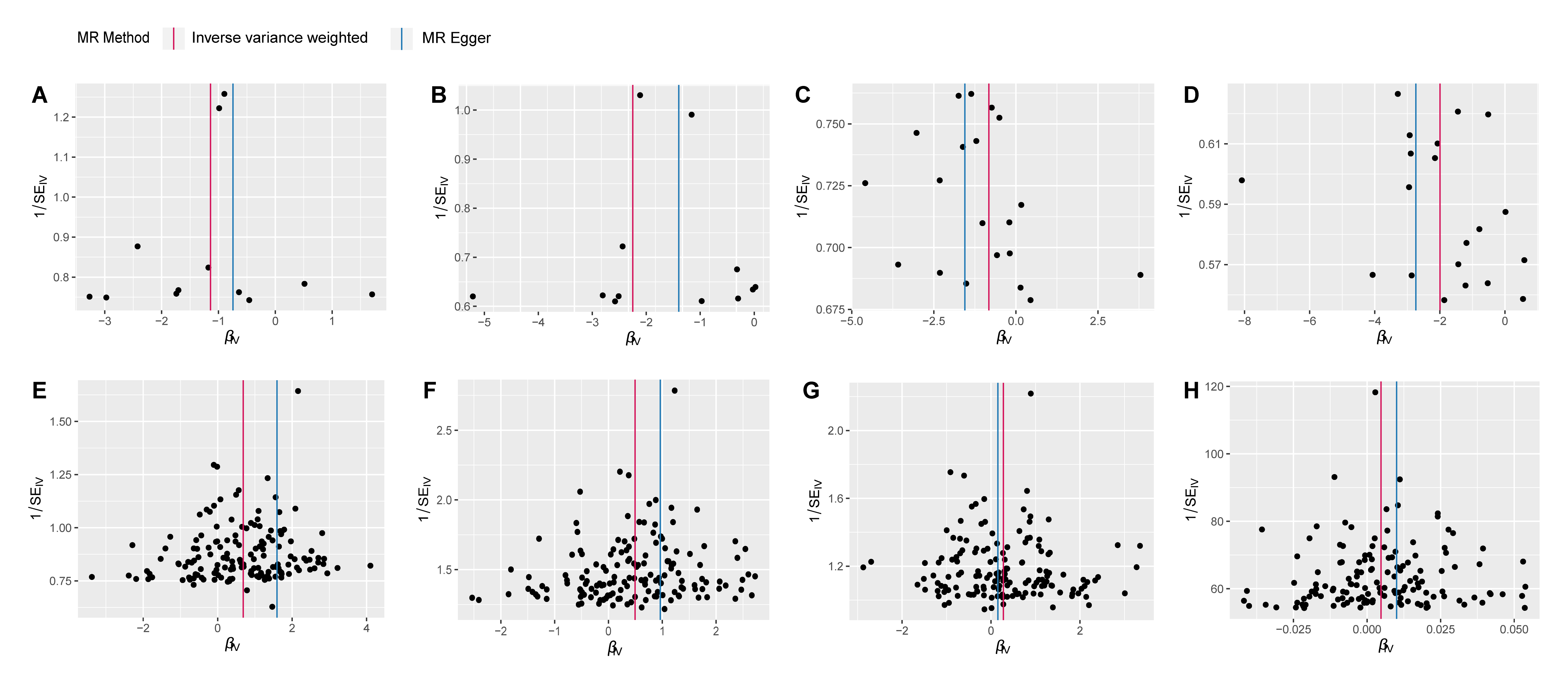

Supplement: Supplementary file 9 — Supplementary Information 9. [file 41598_2023_46984_MOESM9_ESM.tif]
