# Supplementary material for: Causal effect of physical activity and sedentary behaviors on the risk of osteoarthritis: a univariate and multivariate Mendelian randomization study
Source: Sci Rep. 2023 Nov 8;13:19410. doi: 10.1038/s41598-023-46984-2 (PMC10632381; doi:10.1038/s41598-023-46984-2)
Supplement: Supplementary file 10 — Supplementary Figures. [file 41598_2023_46984_MOESM10_ESM.docx]

**Supplemental figure legends**

**Supplemental Figure. 1**. Leave-one-out analysis diagram of the causal relationship between PA and OA risk in MR analysis. The X-axis corresponds to the log (ratio) effect of exposure on outcome. A–B, light DIY on the risk of overall OA and knee OA; C-D, walking for pleasure on the risk of overall OA and knee OA. PA, physical activity; OA, osteoarthritis.

**Supplemental Figure. 2**. Leave-one-out analysis diagram of the causal relationship between SBs and OA and total joint arthroplasty. A–D, television watching on the risk of overall OA, knee OA, hip OA, and TKA. SBs, sedentary behaviors; OA, osteoarthritis; TKA, total knee arthroplasty.

**Supplemental Figure. 3.** Forest plots of PA-associated SNPs on OA risk in the SingleSNP test. A–B, light DIY on the risk of overall OA and knee OA; C-D, walking for pleasure on the risk of overall OA and knee OA. PA, physical activity; OA, osteoarthritis.

**Supplemental Figure. 4**. Forest plots of SBs-associated SNPs on OA and total joint arthroplasty risk in the SingleSNP test. A–D, television watching on the risk of overall OA, knee OA, hip OA, and TKA. SBs, sedentary behaviors; OA, osteoarthritis; TKA, total knee arthroplasty.

**Supplemental Figure. 5**. Funnel plots for the causal estimates of PA/SBs on OA and total joint arthroplasty. Each dot represents an SNP as a genetic tool. A-B, light DIY on the risk of overall OA and knee OA; C-D, walking for pleasure on the risk of overall OA and knee OA; E-H, television watching on the risk of overall OA, knee OA, hip OA, and TKA. PA, physical activity; SBs, sedentary behaviors; OA, osteoarthritis; TKA, total knee arthroplasty.
